# Cadmium-Induced Hydrogen Sulfide Synthesis Is Involved in Cadmium Tolerance in *Medicago sativa* by Reestablishment of Reduced (Homo)glutathione and Reactive Oxygen Species Homeostases

**DOI:** 10.1371/journal.pone.0109669

**Published:** 2014-10-02

**Authors:** Weiti Cui, Huiping Chen, Kaikai Zhu, Qijiang Jin, Yanjie Xie, Jin Cui, Yan Xia, Jing Zhang, Wenbiao Shen

**Affiliations:** 1 College of Life Sciences, Laboratory Center of Life Sciences, Nanjing Agricultural University, Jiangsu Province, Nanjing, China; 2 Key Laboratory of Protection and Development Utilization of Tropical Crop Germplasm Resources, Hainan University, Haikou, China; Key Laboratory of Horticultural Plant Biology (MOE), China

## Abstract

Until now, physiological mechanisms and downstream targets responsible for the cadmium (Cd) tolerance mediated by endogenous hydrogen sulfide (H_2_S) have been elusive. To address this gap, a combination of pharmacological, histochemical, biochemical and molecular approaches was applied. The perturbation of reduced (homo)glutathione homeostasis and increased H_2_S production as well as the activation of two H_2_S-synthetic enzymes activities, including _L_-cysteine desulfhydrase (LCD) and _D_-cysteine desulfhydrase (DCD), in alfalfa seedling roots were early responses to the exposure of Cd. The application of H_2_S donor sodium hydrosulfide (NaHS), not only mimicked intracellular H_2_S production triggered by Cd, but also alleviated Cd toxicity in a H_2_S-dependent fashion. By contrast, the inhibition of H_2_S production caused by the application of its synthetic inhibitor blocked NaHS-induced Cd tolerance, and destroyed reduced (homo)glutathione and reactive oxygen species (ROS) homeostases. Above mentioned inhibitory responses were further rescued by exogenously applied glutathione (GSH). Meanwhile, NaHS responses were sensitive to a (homo)glutathione synthetic inhibitor, but reversed by the cotreatment with GSH. The possible involvement of cyclic AMP (cAMP) signaling in NaHS responses was also suggested. In summary, LCD/DCD-mediated H_2_S might be an important signaling molecule in the enhancement of Cd toxicity in alfalfa seedlings mainly by governing reduced (homo)glutathione and ROS homeostases.

## Introduction

Cadmium (Cd) contamination is a non-reversible accumulation process, with the estimated half-life and high plant-soil mobility, thus resulting in a serious threat to human health through food chains. Normally, Cd exposure leads to the inhibition of plant growth, decrease of crop yield, and even plant cell death [1], [2]. Indirectly stimulated generation of reactive oxygen species (ROS) that modify the antioxidant defence and bring out oxidative stress is ascribed to one of the Cd toxicities in plants, and therefore lipid peroxidation is considered as a hallmark of Cd exposure [3].

In plants, there are a lot of antioxidant defence mechanisms, which could keep the normally formed ROS at a low level and prevent them from exceeding toxic thresholds [3], [4]. The glutathione (GSH) and ascorbate were subsequently recognized as the heart of the redox hub [5]. In plants, GSH is synthesized by two ATP-dependent steps: γ-glutamylcysteine (γ-EC) is synthesized from _L_-glutamate and _L_-cysteine by γ-glutamyl cysteine synthetase (γ-ECS, also called as γ-GCS); and the second step, glycine is conjunct to γ-EC by glutathione synthetase (GS) [6], [7]. In soybean and alfalfa plants, GSH homolog homoglutathione (hGSH) synthesized by homoglutathione synthetase (hGS) from β-alanine and γ-EC, is more abundant than GSH [8]. The rate of glutathione reductase (GR) reaction was the same with either oxidized glutathione (GSSG) or oxidized homoglutathione (hGSSGh) as the substrate [7]. Upon Cd exposure, it was confirmed that the rapid accumulation of peroxides and depletion of GSH and hGSH causes redox imbalance in *Medicago sativa* [9]. Subsequent experiments with comparing ten pea genotypes showing that, activities of ascorbate peroxidase (APX) decreased, but concentrations of GSH increased in the less Cd-sensitive genotypes [10].

Another sulphur-containing compound, hydrogen sulfide (H_2_S), previously known as a toxic gas, has been progressively recognized as a gaseous signaling molecule with multiple functions in animals [11], [12]. For example, H_2_S has been revealed as a cytoprotectant and a regulator in various biological processes, such as oxidative stress suppression, smooth muscle relaxation, proliferation inhibition and apoptosis triggering [13]–[16]. Meanwhile, although previous reports observed that many plants can emit H_2_S [17]–[19], there have been few studies on the physiological role of H_2_S in *planta* during the last century.

In mammals, the majority of endogenous H_2_S was produced by two enzymes, cystathionine *β*-synthase (CBS, EC 4.2.1.22) and cystathionine *γ*-lyase (CSE, EC 4.4.1.1), from _L_-cysteine [20]. Cysteine-degrading enzymes such as cysteine desulfhydrases are hypothesized to be involved in H_2_S release in plants [21]. Previously, two specific desulfhydrases, _L_-cysteine desulfhydrase (LCD, EC 4.4.1.1; also called L-CDes or L-DES) and _D_-cysteine desulfhydrase (DCD, EC 4.4.1.15; also called D-CDes or D-DES), have been isolated and partially analyzed from *Arabidopsis thaliana* [22]–[24]. The LCD, which is considered as the most important enzyme with H_2_S production in plants, shares a 100% sequence homolog with CSE in mammals [25]. By using sodium hydrosulfide (NaHS) as a H_2_S donor, ample evidence further suggested that H_2_S can protect plants against various stress-induced damage, such as salinity stress [26], drought [27]–[29], heavy metal exposure [30], [31], and heat shock [32]. Additionally, H_2_S can act as an inducer in several developmental processes, including adventitious root formation [33] and flower senescence [34]. However, exogenously applied H_2_S donor without checking the kinetics of H_2_S synthesis including corresponding metabolic enzyme activities or transcripts, may not fully replicate the function of endogenous H_2_S in plants.

Cyclic AMP (adenosine 3′, 5′-cyclic monophosphate, cAMP) is a well-known second messenger playing important roles in many physiological processes. The cAMP is synthesized by adenylyl cyclase and broken down by cNMP phosphodiesterase. Dedioxyadenosine (DDA) and 1,3-diazinane-2,4,5,6-tetrone (alloxan) are well characterized as the inhibitors of adenylyl cyclase. Likewise, cNMP phosphodiesterase is sensitive to the inhibitor 1-methyl-3-(2-methylpropyl)-7*H*-purine-2,6-dione (IBMX) [35], [36]. In animals, there is ample evidences to show H_2_S-activited cAMP level or H_2_S-regulated cAMP homeostasis [37], [38]. It was found that H_2_S acted via cAMP-mediated PI3K/Akt/p70S6K signal pathways to inhibit hippocampal neuronal apoptosis and protect neurons from OGD/R-induced injury [39]. However, the functions of cAMP signaling in H_2_S-alleviated Cd stress in plants are still poorly understood.

Thus, the aim of this study was to investigate the signaling role of endogenous H_2_S in the tolerance of *Medicago sativa* seedlings to Cd stress. For this purpose, we preliminarily investigated the synthesis of endogenous H_2_S under Cd stress, which has not been fully performed. Furthermore, the effects of H_2_S on GSH and hGSH metabolism, as well as ROS homeostasis were checked. Our results further indicated that Cd stress triggered endogenous H_2_S production catalyzed by LCD/DCD pathways, and the elevated H_2_S acts as a signal improving the homeostasis of GSH pool and keeping ROS under control, both of which finally contributed to Cd tolerance. Finally, the possible involvement of cAMP signaling in NaHS responses was also suggested.

## Materials and Methods

### Plant material, growth condition

Commercially available alfalfa (*Medicago sativa* L. Victoria) seeds were surface-sterilized with 5% NaClO for 10 min, and rinsed extensively in distilled water before being germinated for 1 d at 25°C in the darkness. Uniform seedlings were then selected and transferred to the plastic chambers and cultured with nutrient medium (quarter-strength Hoagland's solution) in the illuminating incubator (14 h light with a light intensity of 200 µmol·m^−2^·s^−1^, 25±1°C, and 10 h dark, 23±1°C). Five-day-old seedlings were then incubated in quarter-strength Hoagland's solution with or without varying concentrations of NaHS (Sigma-Aldrich; St Louis, MO, USA) or the other indicated chemicals (2 mM _DL_-propargylglycine (PAG), 1 mM GSH, 1 mM _L_-buthionine-sulfoximine (BSO), 50 µM 8-Br-cAMP (8Br), 200 µM alloxan (All), 1 mM DDA, and 500 µM IBMX) alone, or the combination of treatments for 6 h followed by the indicated time points of incubation in 200 µM CdCl_2_. Seedlings without chemicals were used as the control (Con). The pH for both nutrient medium and treatment solutions was adjusted to 6.0.

After various treatments, above-ground parts and root tissues of seedlings were sampled immediately or flash-frozen in liquid nitrogen, and stored at −80°C for further analysis. Among these, above-ground parts and root tissues of 240 seedlings were respectively used for the determination of Cd contents. Seedling root tissues were also used for fresh weight determination (10 seedlings), thiobarbituric acid reactive substances (TBARS) content determination (120 seedlings), and other indicated tests (30 seedlings).

### Determination of H_2_S content, LCD and DCD activity

Hydrogen sulfide content was determined according to the method previously reported [19], [34]. 100 mg of alfalfa seedling roots from 30 seedlings were ground under liquid nitrogen and extracted by 1 ml phosphate buffered saline (50 mM, pH 6.8) containing 0.1 M EDTA and 0.2 M ascorbic acid. After centrifugation at 13000 g for 15 min at 4°C, 400 µl of the supernatant was injected to 200 µl 1% zinc acetate and 200 µl 1 N HCl. After 30 min reaction, 100 µl 5 mM dimethyl-*p*-phenylenediamine dissolved in 7 mM HCl was added to the trap followed by the injection of 100 µl 50 mM ferric ammonium sulfate in 200 mM HCl. After 15 min incubation at room temperature, the amount of H_2_S was determined at 667 nm. Solutions with different concentrations of Na_2_S were used in a calibration curve.

100 mg of alfalfa seedling roots from 30 seedlings were used for activity determination. The activities of LCD and DCD were determined as described by the methods previously reported [23], [40]. _L_-cysteine desulfhydrase (LCD) activity was measured by the release of H_2_S from _L_-cysteine in the presence of dithiothreitol (DTT). The formation of methylene blue was determined at 670 nm. To removal of the background, content of H_2_S in the extracted protein solution was measured by same way with 50% trichloroacetic acid (TCA) instead of _L_-cysteine. The final LCD activity was calculated from the difference between the measured LCD activity and the background. _D_-cysteine desulfhydrase (DCD) activity was measured by the same method with following modifications: _D_-cysteine instead of _L_-cysteine, the pH of Tris-HCl was 8.0 rather than 9.0. Solutions with different concentrations of Na_2_S were prepared, treated in the same way as the assay samples and were used for the quantification of enzymatically formed H_2_S.

### Determination of thiobarbituric acid reactive substances (TBARS), (h)GSH and (h)GSSG(h) contents

Lipid peroxidation was estimated by measuring the amount of TBARS as previously described [41]. About 400 mg of root tissues from 120 seedlings was ground in 0.25% 2-thiobarbituric acid (TBA) in 10% TCA using a mortar and pestle. After heating at 95°C for 30 min, the mixture was quickly cooled in an ice bath and centrifuged at 10,000×*g* for 10 min. The absorbance of the supernatant was read at 532 nm and corrected for unspecific turbidity by subtracting the absorbance at 600 nm. The concentration of lipid peroxides together with oxidatively modified proteins of plants were thus quantified in terms of TBARS amount using an extinction coefficient of 155 mM^−1^ cm^−1^ and expressed as nmol g^−1^ fresh weight (FW).

(h)GSH (GSH + hGSH) and (h)GSSG(h) (GSSG + hGSSGh) contents were measured by the 5,5′dithio-bis-(2-nitrobenzoic acid) (DTNB)-glutathione reductase (GR) recycling assay [41], [42]. Frozen root tissues from 30 seedlings were homogenized in cold 5% 5-sulfosalicylic acid. The homogenate was centrifuged at 12,000×*g* for 20 min at 4°C and the supernatant was collected. Total glutathione ((h)GSH plus (h)GSSG(h)) was determined in the homogenates spectrophotometrically at 412 nm, using GR, DTNB, and NADPH. (h)GSSG(h) contents were determined by the same method in the presence of 2-vinylpyridine and (h)GSH contents were calculated from the difference between total glutathione and (h)GSSG(h).

### Thiol analysis by reversed-phase HPLC

Low-molecular-weight thiols and their corresponding disulfides contents in root tissues from 30 seedlings were measured according to the methods previously reported [43]–[45], through derivatization with monobromobimane (mBBr) after reduction with DTT with or without previously blocked with *N*-ethylmaleimide (NEM), and separation by reversed-phase HPLC (Agilent Technologies, 1200 series Quaternary, Foster city, USA).

### Histochemical analyses

Histochemical detection of lipid peroxidation and loss of plasma membrane integrity was performed with Schiff's reagent and with Evans blue described by previous reports [41], [45].

### Real-time quantitative RT-PCR analysis

Total RNA from root tissues of 30 seedlings was extracted using Trizol reagent (Invitrogen) according to the manufacturer's instructions. DNA-free total RNA (2 µg) from different treatments was used for first-strand cDNA synthesis in a 20-µL reaction volume containing 2.5 units of avian myeloblastosis virus reverse transcriptase XL (TaKaRa) and oligo dT primer.

Real-time quantitative RT-PCR reactions were performed with Mastercycler realplex^2^ real-time PCR system (Eppendorf, Hamburg, Germany) using the SYBR *Premix Ex Taq* (TaKaRa) according to the user manual. The cDNA was amplified using primers (Table S1). The expression levels of the genes are presented as values relative to the corresponding control samples under the indicated conditions, with normalization of data to the geometic average of two internal control genes *MSC27* and *Actin2* [46].

### Visualization of endogenous ROS by LSCM

Endogenous ROS was imaged using the fluorescent probe H_2_DCFDA, and then scanned described by [45], [47].

### Statistical analysis

Values are means ± SD of three different experiments with three replicated measurements. Differences among treatments were analysed by one-way ANOVA, taking *P*<0.05 as significant according to Duncan's multiple range test.

## Results

### (h)GSH depletion and increased endogenous H_2_S synthesis triggered by Cd stress

Considering alfalfa plants contain a thiol tripeptide homolog, hGSH, instead of or in addition to GSH [8], [9], we detected the concentrations of GSH and hGSH. As shown in Table 1, the content of hGSH in alfalfa seedling roots under the control conditions, was about 8-fold higher than that of GSH. Similarly, hGSSGh is the main component of (h)GSSG(h) (total of hGSSGh and GSSG), because the GSSG content was almost negligible.

**Table 1:** Concentrations of low molecular weight thiols and their disulfides, and hGSH/hGSSGh ratio in root tissues.

Treatment	cysteine (nmol g^−1^ FW)	cysteine disulfide (nmol g^−1^ FW)	γ-EC (nmol g^−1^ FW)	γ-EC disulfide (nmol g^−1^ FW)	GSH (nmol g^−1^ FW)	GSSG (nmol g^−1^ FW)	hGSH (nmol g^−1^ FW)	hGSSGh (nmol g^−1^ FW)	hGSH/hGSSGh
Con→Con	30±1 d	3.8±0.8 c	10±1 e	1.5±0.1	27±2 bc	0.2±1.9	252±16 b	28±2 c	8.86
Con→Cd	33±1 cd	5.7±0.8 b	14±2 d	1.7±0.1	21±2 c	0.2±1.4	112±13 f	33±1 bc	3.41
NaHS→Cd	40±2 c	4.0±0.6 c	18±2 bc	1.4±0.5	26±4 c	0.1±1.4	163±14 de	33±4 bc	4.89
NaHS→Con	34±2 cd	3.4±0.7 c	8±0 e	1.2±0.5	36±1 b	0.2±1.1	309±14 a	30±2 bc	10.23
NaHS + PAG→Cd	54±7 b	4.3±0.4 bc	21±3 b	1.2±0.5	29±5 bc	0.3±0.7	144±8 e	41±6 a	3.55
NaHS + PAG + GSH→Cd	65±8 a	4.7±1.6 bc	27±1 a	1.4±0.5	46±11 a	0.7±0.6	179±7 d	36±5 ab	4.91
PAG→Cd	52±6 b	7.4±0.7 a	21±1 b	1.6±0.2	29±1 bc	0.9±0.9	82±12 g	33±3 bc	2.41
PAG→Con	56±4 b	4.0±0.3 c	17±3 cd	1.2±0.5	29±2 bc	0.7±0.7	206±28 c	36±2 ab	5.67

Seedlings were pretreated with or without 100 µM NaHS, 2 mM PAG, 1 mM GSH, individual or combination for 6 h, and then exposed to 200 µM CdCl_2_ for another 12 h. Values are means ± SD of three independent experiments with three replicates for each. Different letters within columns indicate significant differences (*P*<0.05) according to Duncan's multiple range test.

**Figure 1:**
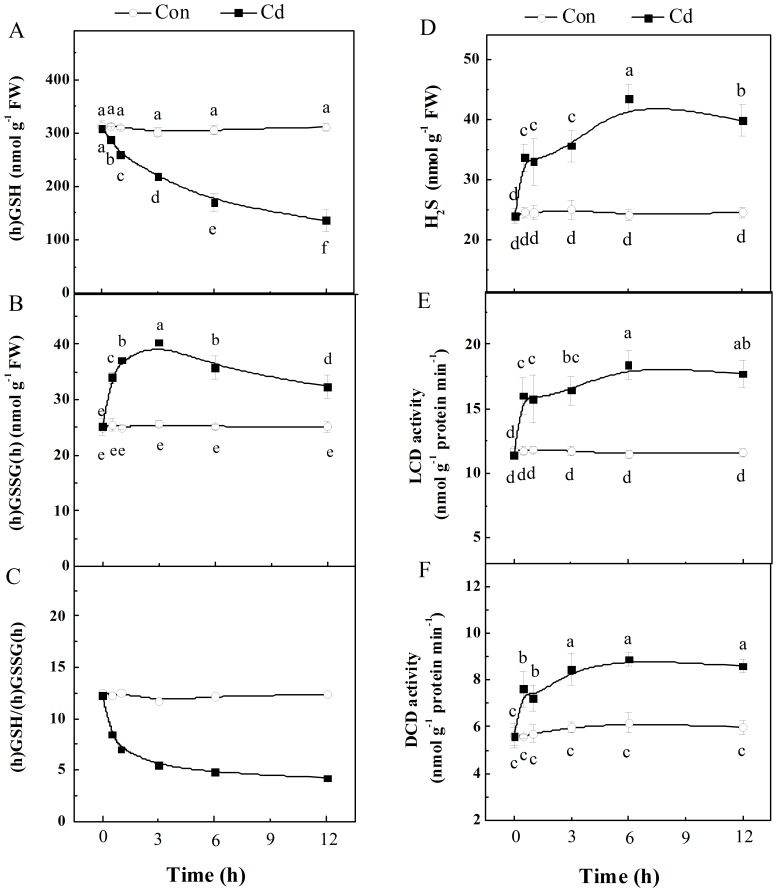
Time course changes of GSH pool and H_2_S synthesis upon Cd stress. Upon 200 µM CdCl_2_ treatment for 12 h, contents of (h)GSH (A), (h)GSSG(h) (B) and H_2_S (D), the ratio of (h)GSH/(h)GSSG(h) (C), and the activities of LCD (E) and DCD (F) in root tissues were analyzed. Values are means ± SD of three independent experiments with three replicates for each. Bars denoted by the same letter did not differ significantly at *P*<0.05 according to Duncan's multiple range test.

To further elucidate the correlation among GSH pool, H_2_S and Cd tolerance, the time course of (homo)glutathione ((h)GSH; total of hGSH and GSH, and (h)GSSG(h)) contents, and H_2_S synthesis were investigated in alfalfa seedling roots upon Cd stress. As expected, a decrease of (h)GSH content (especially hGSH) and an increase of (h)GSSH(h) (especially hGSSGh) level were progressively triggered by Cd stress within 12 h, thus leading to a decreased (h)GSH/(h)GSSH(h) ratio (12 h; Figure 1A-C), an important parameter for the intracellular redox status in *planta* upon Cd stress [3], [45]. The ratio of hGSH/hGSSGh exhibited the similar tendency (Table 1). These results were consistent with the observed Cd toxicity, confirmed by the histochemical staining detecting the aggravated loss of plasma membrane integrity and lipid peroxidation with Evans blue and Schiff's reagent, increased TBARS content and growth stunt of seedling roots (Figure S1).

Because H_2_S synthesis could be induced by oxidative stress and depletion of GSH both in animals and plants [48]–[50], we simultaneously investigated the production of H_2_S in seedling roots after the exposure to Cd. Similar to the recent report [51], the production of H_2_S was continuously increased after the exposure to Cd alone for 12 h (Figure 1D). The changes in activities of two H_2_S synthetic enzymes LCD and DCD displayed similar tendencies (Figure 1E and F). Apparently, the reduced (homo)glutathione depletion and increased endogenous H_2_S synthesis preceded Cd toxicity in alfalfa seedlings.

### NaHS not only mimics intracellular H_2_S content, but also alleviates Cd toxicity

Previous results revealed that the exogenously applied NaHS, a H_2_S donor, alleviates Cd toxicity in bermudagrass seedlings [51]. Therefore, a preliminary work was carried out to compare the oxidative damage and growth performance of alfalfa seedlings upon Cd exposure with or without the indicated concentrations of NaHS pretreatment. Firstly, the results of histochemical staining and TBARS contents revealed that NaHS at 100 (in particular) and 500 µM was able to significantly decreased Cd-induced lipid peroxidation (Figure S1A and B). These beneficial roles were also supported by the changes of fresh weight of ten alfalfa seedling roots, showing that NaHS at 100 and 500 µM had the greatest effects on the alleviation of the inhibition of root growth caused by Cd stress (Figure S1C). The beneficial roles of 100 µM NaHS alone were also observed. Subsequent work confirmed that H_2_S rather than other sulphur-containing derivatives and sodium exhibited the cytoprotective role in the improvement of Cd toxicity by using a series of sulphur- and sodium-containing chemicals including Na_2_S, Na_2_SO_4_, Na_2_SO_3_, NaHSO_4_, NaHSO_3_, and NaAc, in comparison with the positive roles of NaHS (Figure S2).

**Figure 2:**
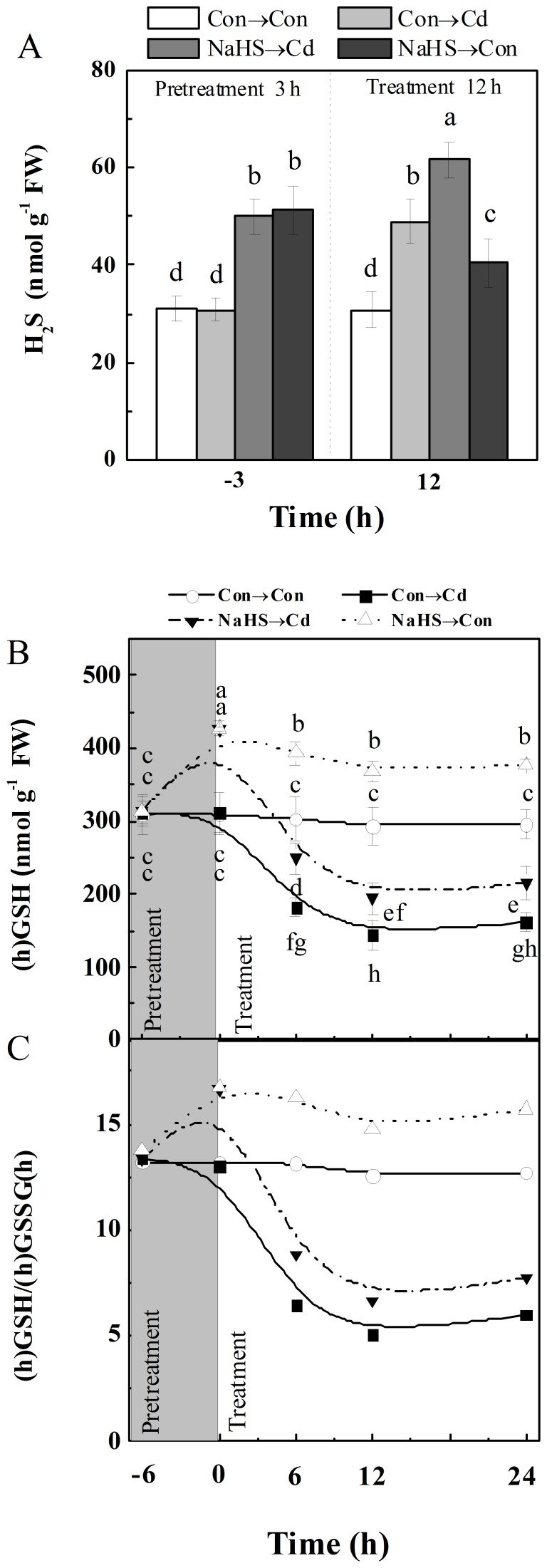
NaHS increased endogenous H_2_S and (h)GSH contents, and the ratio of (h)GSH/(h)GSSG(h) upon Cd stress. Endogenous H_2_S concentration in root tissues (A) was detected at 3 h after the beginning of 100 µM NaHS pretreatment (−3 h), and 200 µM CdCl_2_ or chemical-free control treatments for 12 h (12 h). Meanwhile, contents of (h)GSH (B) and the ratio of (h)GSH/(h)GSSG(h) (C) in root tissues were detected at the indicated time points of treatments. Values are means ± SD of three independent experiments with three replicates for each. Bars denoted by the same letter did not differ significantly at *P*<0.05 according to Duncan's multiple range test.

Accordingly, we observed that the treatment with 100 µM NaHS for 3 h resulted in the enhancement of endogenous H_2_S level in alfalfa seedling roots, which also mimicked a physiological response elicited by Cd alone for 12 h (Figure 2A). The addition of Cd to the NaHS-pretreated plants further strengthened the increased H_2_S content. Therefore, 100 µM NaHS was used to mimic the physiological role of intracellular H_2_S in the subsequent experiments.

### Changes of low molecular weight thiols and their disulfides as well as representative transcripts in response to NaHS

To determine the influence of H_2_S at physiologically concentrations on (h)GSH depletion, GSH pool and corresponding metabolism associated genes were investigated. As shown in Figure 2B, the time-course analysis revealed that (h)GSH contents in seedling roots were significantly enhanced by the pretreatment with NaHS for 6 h, and remained high through 24 h of further incubation in the control solution. Meanwhile, NaHS pretreatment was able to slow down the decreased (h)GSH levels caused by Cd exposure. Changes of the (h)GSH/(h)GSSG(h) ratio also exhibited the similar tendencies (Figure 2C). Comparatively, Cd-induced cysteine and γ-EC (in particular), and cysteine disulfide contents were differentially strengthened or blocked by NaHS pretreatment, respectively (Table 1).

**Figure 3:**
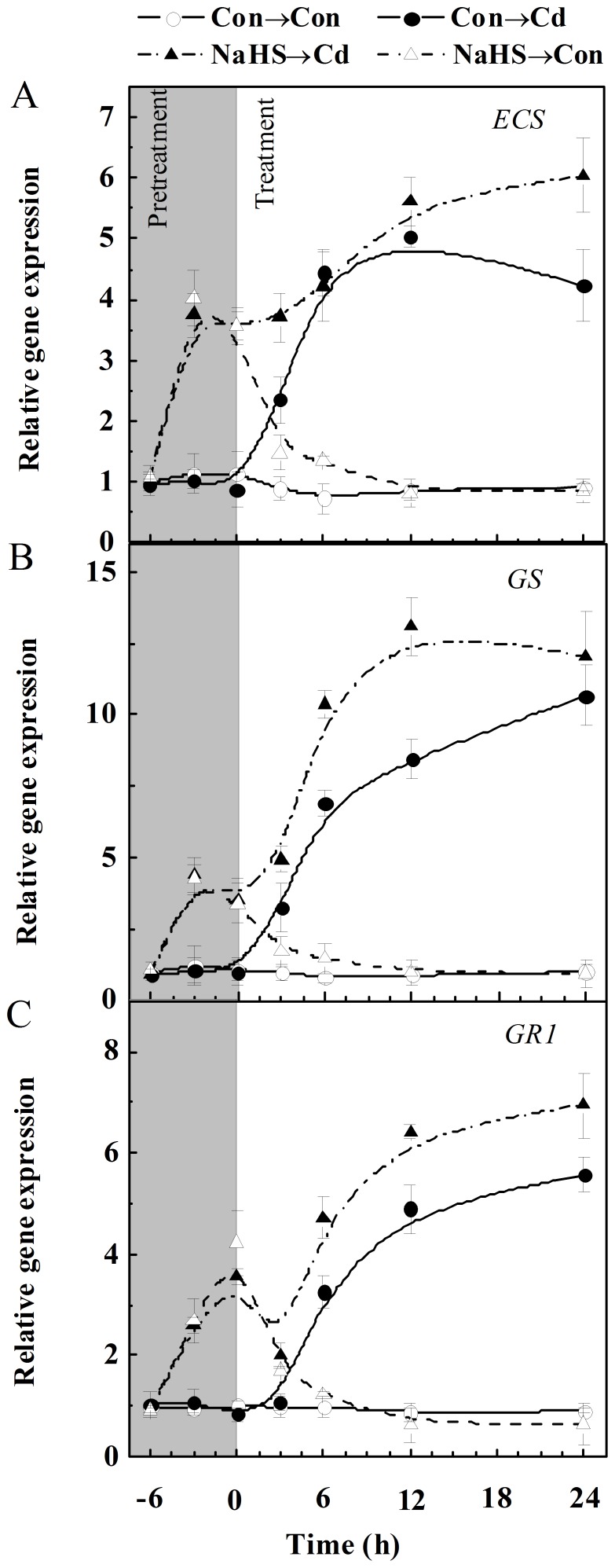
Time course of transcripts responsible for (h)GSH metabolism regulated by NaHS and Cd. Seedlings were pretreated with or without 100 µM NaHS for 6 h and then exposed to 200 µM CdCl_2_ for another 24 h. The expression levels of *ECS* (A), *GS* (B) and *GR1* (C) in root tissues analyzed by real-time RT-PCR are presented as values relative to the control at the beginning of pretreatment, normalized against expression of two internal reference genes in each sample. Values are means ± SD of three independent experiments with three replicates for each.

These results arises the question that, whether this increases in metabolites are, at least in part, duo to changes in the expression of genes involved in (h)GSH metabolism. Therefore, the expression of *ECS*, *GS*, and *GR1* genes, were analyzed by real-time RT-PCR. Results of Figure 3A and B revealed that the transcripts of *ECS*, *GS* and *GR1* (especially) in seedling roots approximately displayed a time-dependent increase during Cd stress for 24 h, while the transcriptional profiles of these genes in the control samples were relatively constant during the same period. The pretreatment with NaHS for 6 h in culture solution increased above transcripts, which were differentially strengthened by thereafter Cd stress.

### NaHS-induced Cd tolerance, (h)GSH and ROS homeostases were sensitive to PAG, but rescued by GSH

**Figure 4:**
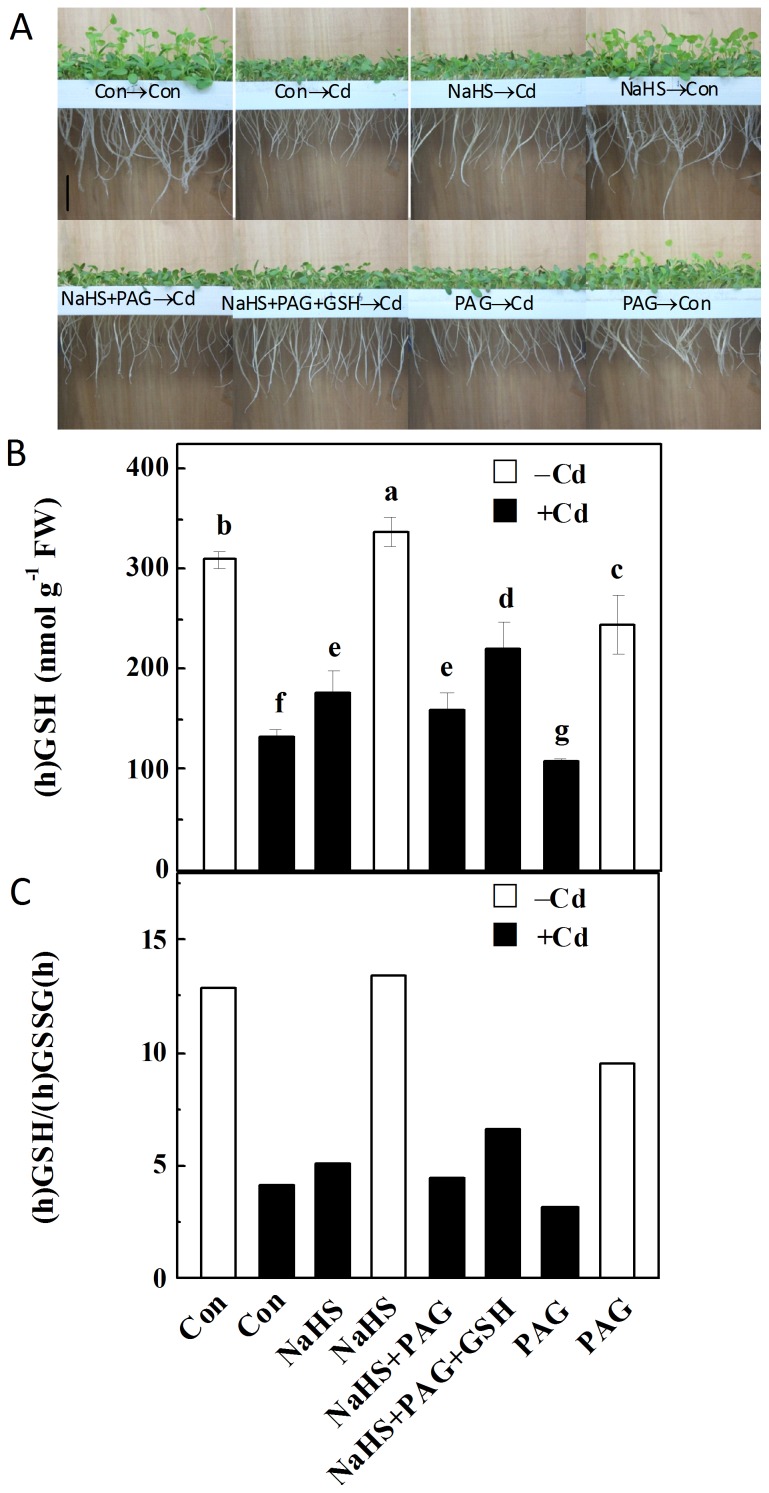
NaHS, PAG and GSH pretreatments differentially regulated seedling growth, (h)GSH content, and (h)GSH/(h)GSSG(h) ratio. Corresponding phenotypes were photographed after 200 µM CdCl_2_ treatment for 72 h, with or without 100 µM NaHS, 2 mM PAG, 1 mM GSH, individual or combination pretreatments for 6 h (A). Scale bar, 2 cm. Contents of (h)GSH (B), and the ratio of (h)GSH/(h)GSSG(h) (C) in root tissues were also analyzed after 200 µM CdCl_2_ treatment for 12 h, with or without 100 µM NaHS, 2 mM PAG, 1 mM GSH, individual or combination pretreatment for 6 h. Values are means ± SD of three independent experiments with three replicates for each. Bars denoted by the same letter did not differ significantly at *P*<0.05 according to Duncan's multiple range test.

To further verify the involvement of endogenous H_2_S in Cd tolerance, _DL_-propargylglycine (PAG), an effective H_2_S synthetic inhibitor [27], and GSH, applied individually and in combination, were used in the subsequent experiment. After 72 h exposure to Cd, the alfalfa seedlings displays severe growth inhibition both in roots and above ground parts, compared to control samples, both of which were improved by NaHS pretreatment (Figure 4A). By contrast, the improvement of seedling growth inhibition as well as the reestablishment of (h)GSH homeostasis triggered by NaHS were sensitive to PAG, but blocked by exogenously applied GSH (Figure 4, Figure S3A). An aggravated Cd toxicity in seedling growth inhibition was also observed when PAG was pretreated.

**Figure 5:**
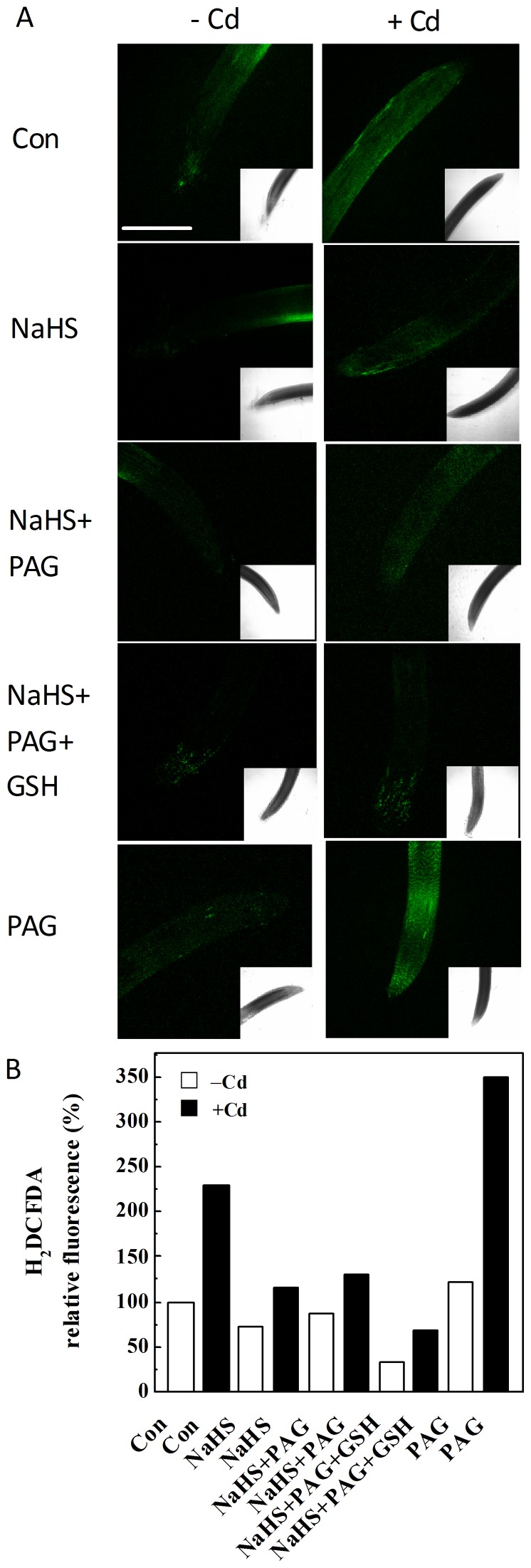
NaHS and GSH pretreatments alleviated Cd-induced ROS production, but blocked by PAG. LSCM results (A). Seedlings were pretreated with or without 100 µM NaHS, 2 mM PAG, 1 mM GSH, individual or combination for 6 h, and then exposed to 200 µM CdCl_2_ for another 6 h. After various treatments, the roots were respectively stained with H_2_DCFDA, then washed thoroughly to removal extra dye and immediately photographed by LSCM. Scale bar, 0.5 mm. The relative DCF fluorescence intensity in the corresponding roots (B).

In an attempt to assess the potential role of endogenous H_2_S in ROS homeostasis in Cd-stressed seedlings, ROS production was visualized by staining with H_2_DCFDA. As expected, ROS in root tips with Cd alone were produced considerably, suggesting a perturbation in ROS homeostasis (Figure 5). However, the pretreatment with NaHS reduced the ROS abundance. Further results revealed that PAG pretreatment increased the H_2_DCFDA fluorescence in Cd-stressed seedling roots, which was further blocked by the addition of GSH. The changes of TBARS content, an indictor of lipid peroxidation, exhibited the similar tendencies (Figure S3B).

Cd treatment caused the accumulation of Cd contents both in shoot and root (particularly) tissues (Figure S4). Similar to the previous reports [31], NaHS decreased Cd accumulation, which was significantly reversed by PAG, but was further blocked by the cotreatment with GSH.

### Transcripts of representative antioxidant defense genes were sensitive to PAG, but rescued by GSH

**Figure 6:**
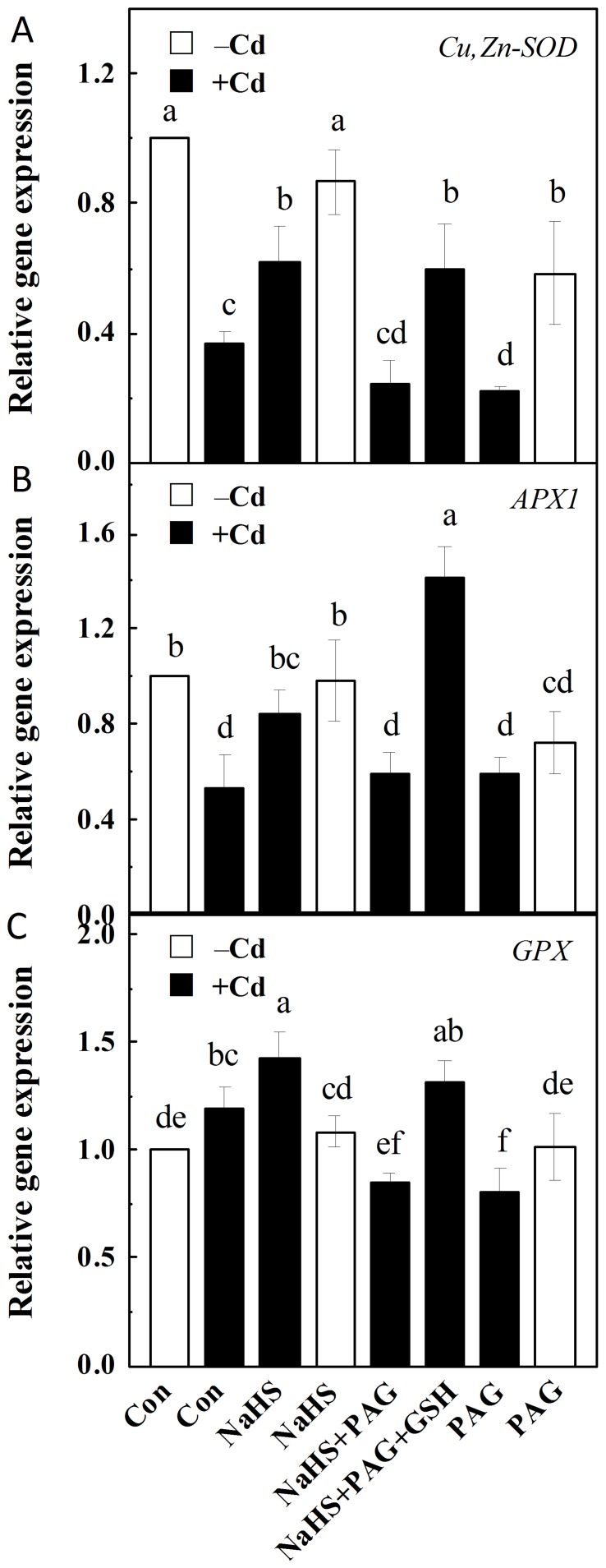
Transcripts of *Cu, Zn-SOD*, *APX1*, and *GPX* regulated by NaHS, PAG, GSH and Cd. Seedlings were pretreated with or without 100 µM NaHS, 2 mM PAG, 1 mM GSH, individual or combination for 6 h, and then exposed to 200 µM CdCl_2_ for another 12 h. The expression levels of *Cu,Zn-SOD* (A), *APX1* (B), and *GPX* (C) transcripts in root tissues analyzed by real-time RT-PCR are presented as values relative to the control, normalized against expression of two internal reference genes in each sample. Values are means ± SD of three independent experiments with three replicates for each. Bars denoted by the same letter did not differ significantly at *P*<0.05 according to Duncan's multiple range test.

Since ROS homeostasis was reestablished by NaHS in stressed conditions, the real-time RT-PCR test of corresponding genes involved in their metabolism, i.e. *Cu*, *Zn-SOD*, *APX1*, and *GPX* [3], [5], were analysed. The results of Figure 6 revealed that in comparison with Cd alone samples, NaHS pretreatment followed by Cd exposure resulted in the enhancement in the transcript levels of *Cu*, *Zn-SOD*, *APX1*, and *GPX* in alfalfa seedling roots. The addition of PAG, however, significantly blocked the increases in the transcripts levels of these representative antioxidant enzymes induced by NaHS, all of which were reversed when GSH was added together with PAG.

### NaHS responses were sensitive to a (h)GSH synthetic inhibitor, but reversed by the added GSH

**Figure 7:**
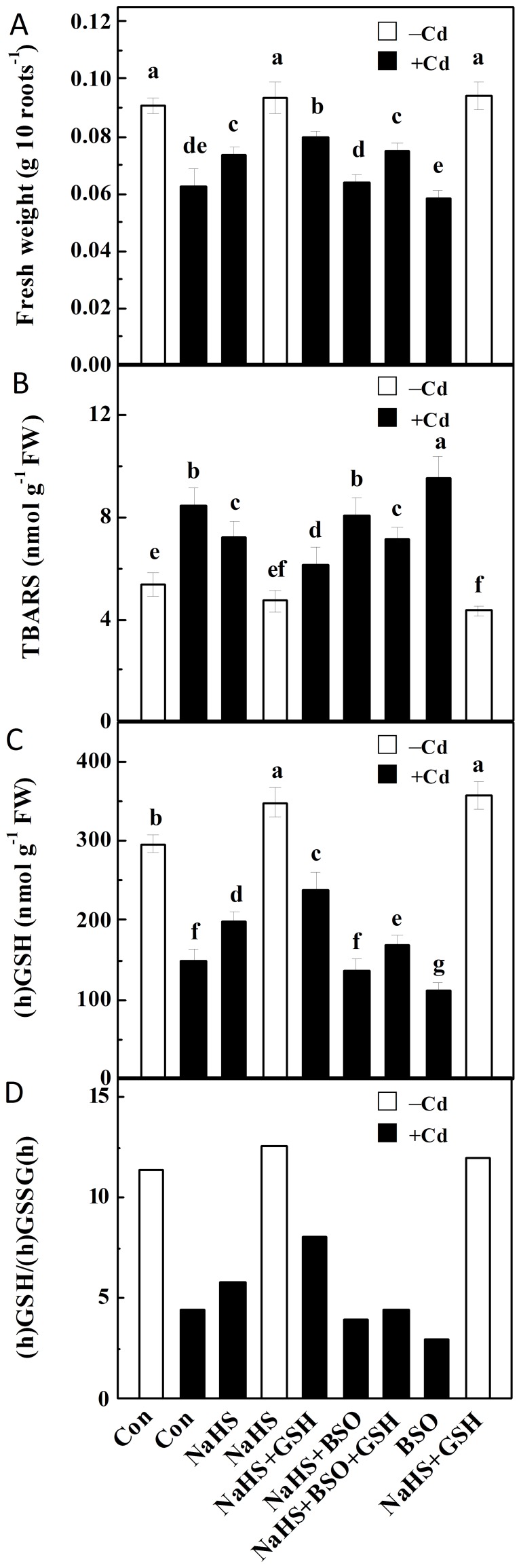
NaHS, GSH and BSO pretreatments differentially regulated seedling growth, TBARS accumulation, (h)GSH content, and (h)GSH/(h)GSSG(h). Fresh weight of 10 roots (A), TBARS accumulation (B), (h)GSH contents (C), and (h)GSH/(h)GSSG(h) ratio (D) in root tissues were determined after seedlings were pretreated with or without 100 µM NaHS, 1 mM GSH, 1 mM BSO, individual or combination for 6 h, and then exposed to 200 µM CdCl_2_ for 72 h (A), 24 h (B) and 12 h (C and D). Values are means ± SD of three independent experiments with three replicates for each. Bars denoted by the same letter did not differ significantly at *P*<0.05 according to Duncan's multiple range test.

The involvement of (h)GSH homeostasis in NaHS-induced cytoprotective against Cd stress were further investigated using a (h)GSH synthetic inhibitor and GSH applied exogenously. Pretreatment with NaHS, and _L_-buthionine-sulfoximine (BSO) at 1 mM, a concentration expected to be effective [52], exhibited an aggravated Cd toxicity, which was confirmed by the severe growth stunt and TBARS overproduction, in comparison with Cd plus NaHS (Figure 7A and B). Similarly, NaHS-mediated reestablishment of (h)GSH homeostasis in Cd stressed alfalfa seedling roots was also perturbed by BSO (Figure 7C and D), which was confirmed by the significant decreased (h)GSH content and the ratio of (h)GSH/(h)GSSG(h), respect to Cd alone. By contrast, above BSO responses were sensitive to the addition of GSH when applied together. Above results clearly indicated a requirement for (h)GSH homeostasis in NaHS-mediated alleviation of Cd toxicity.

### cAMP signaling might be involved in NaHS responses

**Figure 8:**
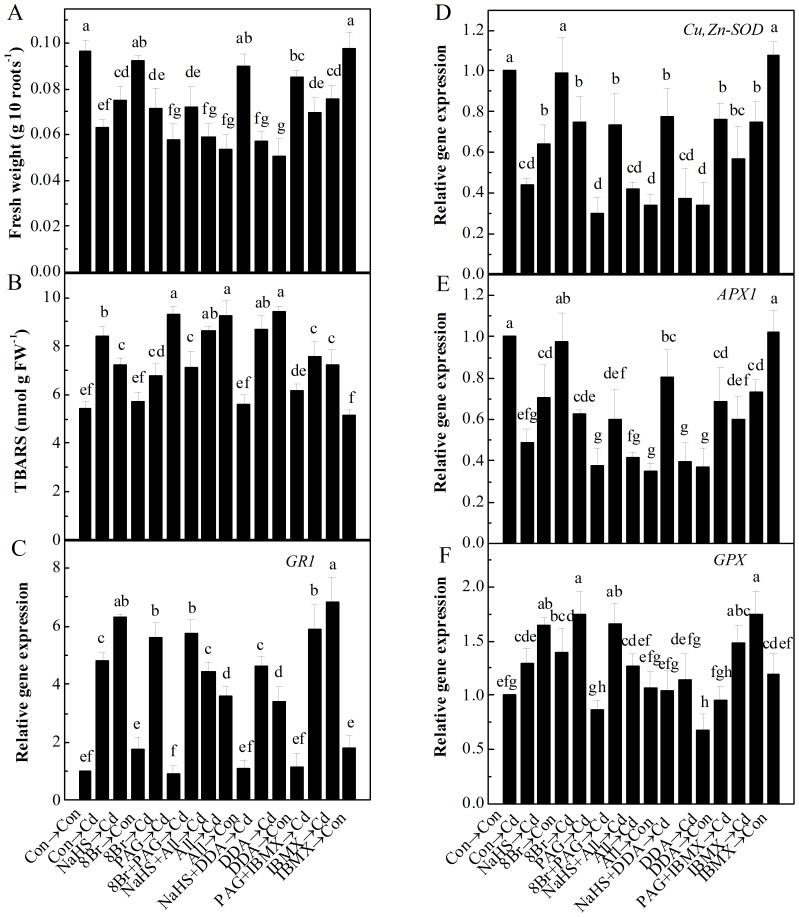
cAMP pathway might be involved in H_2_S-alleviated Cd toxicity. Fresh weight of 10 roots (A), TBARS accumulation (B), *GR1* (C), *Cu,Zn-SOD* (D), *APX1* (E), and *GPX* (F) gene expression in alfalfa seedling roots upon Cd stress. Seedlings were pretreated with or without 100 µM NaHS, 50 µM 8-Br-cAMP (8Br), 2 mM PAG, 200 µM alloxan (All), 500 µM IBMX, 1 mM DDA alone, or the combination of treatments for 6 h, and then exposed to 200 µM CdCl_2_ for 72 h (A), 24 h (B) and 12 h (C–F). The expression levels of corresponding genes analyzed by real-time RT-PCR are presented as values relative to the control, normalized against expression of two internal reference genes in each sample. Values are means ± SD of three independent experiments with three replicates for each. Bars denoted by the same letter did not differ significantly at *P*<0.05 according to Duncan's multiple range test.

To testify the hypothesis that H_2_S response is associated with cAMP signaling pathway, a pharmacological approach was used to manipulate endogenous cAMP. Results presented in Figure 8A and B indicated that the pretreatment with 8-Br-cAMP, a membrane-permeable analogue of cAMP, alleviated Cd-induced decrease of fresh weight and increase of TBARS content in alfalfa seedling roots. Both of two adenylyl cyclase inhibitors, alloxan and DDA, blocked NaHS-alleviated Cd stress. Moreover, similar to the beneficial actions of 8-Br-cAMP (when was cotreated with PAG followed by Cd stress), a cNMP phosphodiesterase inhibitor IBMX also reversed the PAG responses in the aggravation of fresh weight loss and lipid peroxidation caused by Cd stress. Results from the real-time RT-PCR showed that 8-Br-cAMP and IBMX pretreatments followed by Cd stress, mimicked the effect of NaHS on *GR1* up-regulation, regardless of whether PAG was added or not (Figure 8C). Two inhibitors alloxan and DDA partially blocked NaHS plus Cd-induced *GR1* transcripts. A similar tendency was found in the changes in *GPX* transcripts (Figure 8F). Results presented in Figure 8D and E further revealed the negative effects of adenylyl cyclase inhibitors on the transcripts of *Cu*, *Zn-SOD* and *APX1* in NaHS-pretreated seedling roots upon Cd, in comparison with the positive responses of 8-Br-cAMP and IBMX in the presence or absence of PAG.

## Discussion

Although H_2_S is a hazardous gaseous molecule with a strong odor of rotten eggs, it has been described as an important regulator with a variety of biological roles in animals and recently in plants [11]–[16], [25]–[34], [53]–[56]. Moreover, recent works on *Populus euphratica* cells [57] and bermudagrass seedlings [51], demonstrated that exogenously applied NaHS, a H_2_S donor, resulted in an enhanced Cd tolerance in these species. However, possible physiological mechanisms and downstream targets responsible for the observed Cd tolerance triggered by intracellular H_2_S remain elusive. In this report, we discovered endogenous H_2_S production in response to Cd stress, and further provided evidence demonstrating a requirement of (h)GSH and ROS homeostases, at least partially, in the intracellular H_2_S-medaited plant adaptation against Cd toxicity. Therefore, our results presented in this work are vital for both fundamental and applied plant biology.

### Endogenous H_2_S production in response to Cd stress: the possible involvement of LCD/DCD

In animals, it was previously reported that diverse stress-inducing stimuli could result in the production of H_2_S, including oxidative stress [49], depletion of cysteine (or its derivatives) [58] and glutathione [50]. Recent work in Arabidopsis [25] and bermudagrass seedlings [51] reported drought- and Cd-induced H_2_S production. Because the signal compound H_2_S is very reactive [53], the rapid regulation of the activity of H_2_S biosynthetic enzymes seems essential to fulfill H_2_S-depenent functions. In this work, we further showed that Cd-triggered endogenous H_2_S production might be related to LCD/DCD pathways (Figure 1D-F), since the similar increasing changes in the levels of intracellular H_2_S as well as LCD/DCD activities were observed in the seedling roots of alfalfa challenged with Cd for 12 h. Meanwhile, similar to previous reports in wheat [30], bermudagrass [51], *Spinacia oleracea* seedlings [59], and strawberry plants [60], NaHS-induced H_2_S production in alfalfa plants was also observed (Figure 2A).

In plants, both LCD and DCD are hypothesized to be involved in intracellular H_2_S synthesis [21] [27]. Several LCD/DCD candidates have been cloned and partially analyzed from the model plant *Arabidopsis* to *Brassica napus* [24] [61]. Our above findings are consistent with those reported by Bloem et al. [40], in which they found that *Brassica napus* was able to react to *Pyrenopeziza brassicae* infection with a greater potential to release H_2_S, which was reflected by an increasing LCD activity with fungal infection. More recently, auxin-induced DES-mediated H_2_S generation was also found to be involved in lateral root formation in tomato seedlings [62]. In view of the fact that all H_2_S synthetic enzymes are not fully elucidated, our results suggested that LCD/DCD pathways might be, at least partially, related to Cd-induced H_2_S production in alfalfa seedlings. In a future study, the role of other enzymatic and non-enzymatic pathways-mediated induction of H_2_S synthesis in alfalfa seedlings upon Cd stress need be further elucidated.

### The mechanism underlying the role of intracellular H_2_S in the alleviation of Cd toxicity: reestablishment of reduced (homo)glutathione and ROS homeostases

Ample evidence revealed a clear relationship between metal stress and redox homeostasis and antioxidant capacity [3] [9] [63]–[66]. Also, GSH could function as a heavy metal-ligand and an antioxidant [5] [67]. In plants, H_2_S serves as a signal as well as a novel antioxidant in hormonal and defense responses against abiotic stress [53] [60]. Genetic evidence further revealed that the GSH deficiency mutant *pad2-1* shows the more oxidized redox state in contrast to wild type [68]. Arabidopsis mutants deficient in phytochelatins (PCs) and GSH biosynthesis respectively, *cad1* and *cad2*, are consequently more sensitive to Cd [6] [69] [70], that showed the crucial role of PCs, especially their precursor GSH in responding to Cd challenge. In the assays described here, as expected, when alfalfa seedling plants were upon Cd exposure, (h)GSH homeostasis is altered, which is reflected by the fact that the concentrations of reduced GSH and hGSH dropped (Table 1, Figure 1A–C), possible as a consequence of initiated PCs biosynthesis [71] [72]. Similarly, a low ratio of (h)GSH/(h)GSSH(h), an important redox index related to Cd tolerance in alfalfa plants [45], was also observed. These changes thereafter cause redox imbalance and in turn Cd toxicity (Figures S1A, S3 and S4; Figures 4A and 5).

Our further experiments provide strong evidence to support the existence of a causal relationship between the endogenous H_2_S signal and the alleviation of Cd toxicity in alfalfa seedlings partly by reestablishment of (h)GSH and ROS homeostases, which might be associated with the cAMP pathway. This conclusion is based on several pieces of evidence: (i) increased H_2_S metabolism as well as the perturbation of (h)GSH homeostasis in alfalfa seedling roots are two early responses to the exposure of Cd (Figure 1, Table 1). These changes were consistent with the phenotypes of Cd toxicity (Figure 4A, Figure S1C). (ii) Application of a H_2_S-releasing compound NaHS (also called as H_2_S donor), not only mimics intracellular H_2_S content triggered by Cd, but also alleviates Cd toxicity (Figures 2 and 4). Consistently, we also detected reestablishment of (h)GSH homeostasis, which was reflected by a higher (h)GSH content and ratio of (h)GSH/(h)GSSG(h) upon Cd stress. The observed Cd tolerance might be due to the available (h)GSH by the up-regulation of (h)GSH synthesis related genes, *ECS* and *GS* (Figure 3A and B), as well as *GR1* (Figure 3C), because besides the synthesis of PCs, availability of GSH and concerted activity of GR seem to play a important role for plants to combat oxidative stress and Cd toxicity [7] [72] [73]. While, the inhibition of H_2_S production caused by its synthetic inhibitor PAG blocked NaHS-induced Cd tolerance and reestablishment of (h)GSH and ROS homeostases, the latter of which was confirmed by the histochemical staining detecting the alleviation of plasma membrane integrity and lipid peroxidation, decreased ROS content and up-regulation of *Cu,Zn-SOD*, *APX1* and *GPX* transcripts, as well as declined TBARS level (Table 1, Figures 2–6, and Figures S1, S3 and S4). (iii) Above mentioned PAG responses were further rescued by exogenously applied GSH (Table 1, Figures 4–6). (iv) NaHS responses were sensitive to a (h)GSH synthetic inhibitor, but reversed by the added GSH (Figure 7), both of which suggesting a requirement of (h)GSH homeostasis for NaHS cytoprotective roles; and (v) Previous reports in animals showed H_2_S-activited cAMP level or H_2_S-regulated cAMP homeostasis [37] [38]. Here, we found that two adenylyl cyclase inhibitors, alloxan and DDA, blocked the beneficial responses conferred by NaHS in alfalfa seedlings subjected to Cd stress (Figure 8). On the contrary, an analogue of cAMP 8-Br-cAMP and a cNMP phosphodiesterase inhibitor IBMX mimicked the effects of NaHS on the alleviation of Cd toxicity as well as the regulation of (h)GSH homeostasis and ROS metabolism (*GR1*, *Cu,Zn-SOD*, *APX1*, and *GPX*, etc). Above pharmacological evidence indicated the involvement of cAMP signaling in NaHS responses. Additionally, NaHS-triggered cytoprotective roles were confirmed to act as a H_2_S-dependent fashion (Figure S2). Above results clearly established a casual link between intracellular H_2_S in the alleviation of Cd toxicity and reestablishment of (h)GSH and ROS homeostases.

## Conclusions

**Figure 9:**
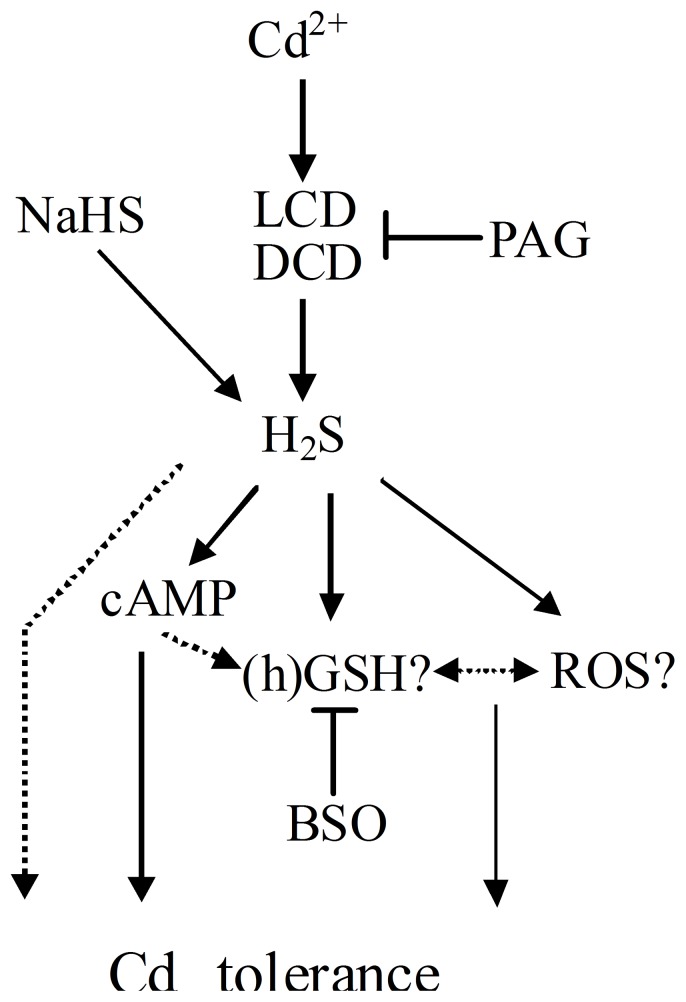
Simplified scheme of mechanisms involved in Cd tolerance by LCD/DCD-produced H_2_S-modulated (h)GSH and ROS homeostases. Abbreviations: NaHS, sodium hydrosulfide; PAG, _DL_-propargylglycine; LCD, _L_-cysteine desulfhydrase; DCD, _D_-cysteine desulfhydrase; H_2_S, hydrogen sulfide; ROS, reactive oxygen species; (h)GSH, reduced (homo)glutathione; BSO, _L_-buthionine-sulfoximine; cAMP, cyclic AMP. The dashed line denotes possible signaling cascade. T bars, inhibition.

In summary, our pharmacological, histochemical, biochemical and molecular evidence suggested that the intracellular H_2_S was able to ameliorate Cd toxicity in alfalfa seedlings at least partly by reestablishment of (h)GSH and ROS homeostases. Figure 9 illustrates a simplified scheme of mechanisms involved in Cd tolerance by LCD/DCD-produced H_2_S-modulated (h)GSH and ROS homeostases, since 1) LCD/DCD-produced H_2_S acts as a signal triggered by Cd to regulated (h)GSH metabolisms; 2) both (h)GSH and ROS homeostases could be reestablished by H_2_S and further linked to Cd tolerance; 3) cAMP signaling pathway might be related to NaHS-triggered Cd tolerance, partially through the regulation of GSH homeostasis and ROS metabolism. Taking into account that H_2_S participates in stressful responses and developmental process, our study therefore may extend our understanding of the complex system integrating environmental and developmental signals.

## Supporting Information

Figure S1 **NaHS pretreatment alleviates Cd toxicity.**
(DOC)

Figure S2 **H_2_S or HS^−^, but not other compounds derived from NaHS contribute to NaHS responses.**
(DOC)

Figure S3 **Effects of NaHS, PAG and GSH pretreatments on the fresh weight (A) and TBARS concentrations (B) in alfalfa seedling roots upon Cd stress.**
(DOC)

Figure S4 **Effects of NaHS, PAG and GSH pretreatments on Cd concentrations in alfalfa seedlings upon Cd stress.**
(DOC)

Table S1 **The sequences of primers for real-time RT-PCR.**
(DOC)
