# Supplementary material for: Cadmium-Induced Hydrogen Sulfide Synthesis Is Involved in Cadmium Tolerance in Medicago sativa by Reestablishment of Reduced (Homo)glutathione and Reactive Oxygen Species Homeostases
Source: PLoS One. 2014 Oct 2;9(10):e109669. doi: 10.1371/journal.pone.0109669 (PMC4183592; doi:10.1371/journal.pone.0109669)
Supplement: Figure S1 — NaHS pretreatment alleviates Cd toxicity. (DOC) [file pone.0109669.s001.doc]

**Supplementary Figure S1**

**Supplementary Figure S1.** **NaHS pretreatment alleviates Cd toxicity.** Five-day-old seedlings were pretreated with or without 0-500 μM NaHS for 6 h, and then exposed to 200 μM CdCl_2_ for 24 h (A and B) or 72 h (C). Afterwards, the roots were histochemically stained with Evan blue and Schiff’s reagent (A) for the determination of plasma membrane integrity and lipid peroxidation (A). Thus, representative pictures were provided. Bar, 0.5 mm. Furthermore, TBARS contents (B) and growth inhibition of 10 seedling roots (C) were also determined. Values are means ± SD of three independent experiments with three replicates for each. Bars denoted by the same letter did not differ significantly at *P* < 0.05 according to Duncan’s multiple range test.


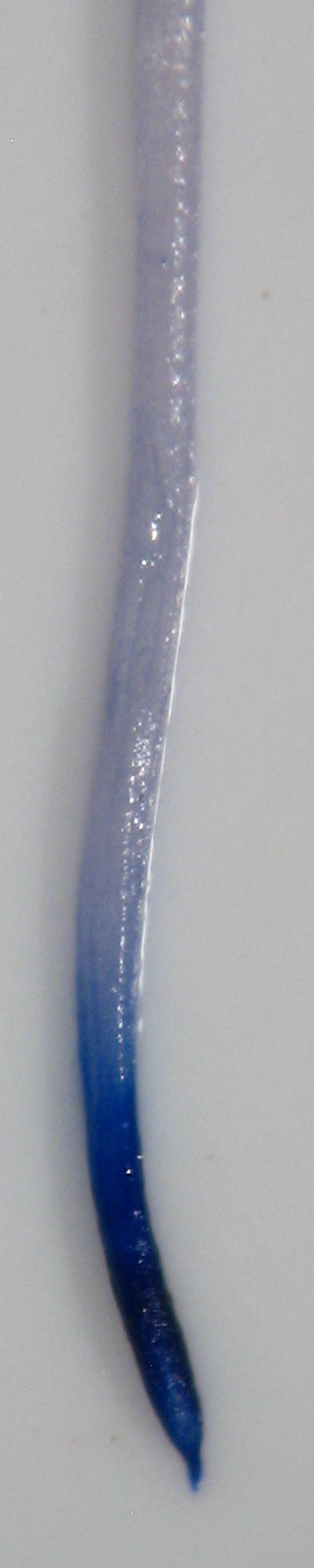

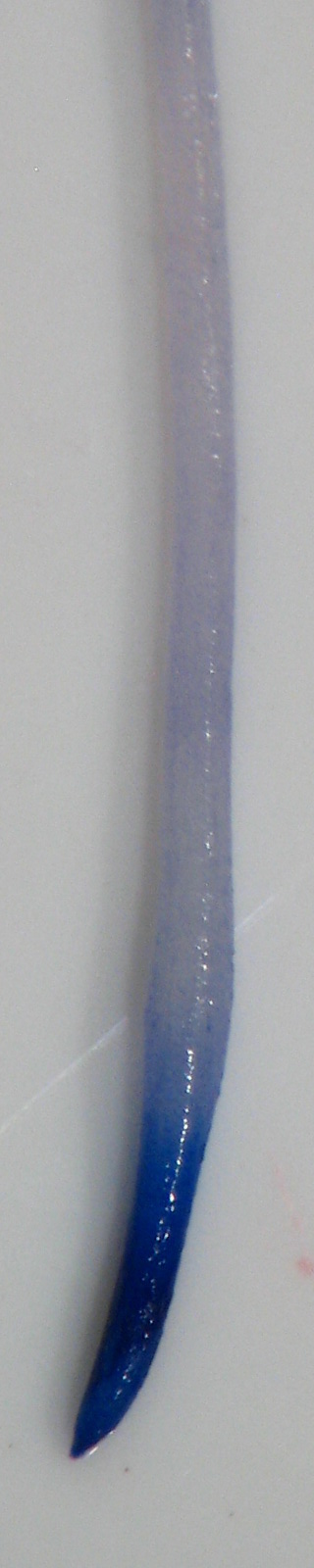

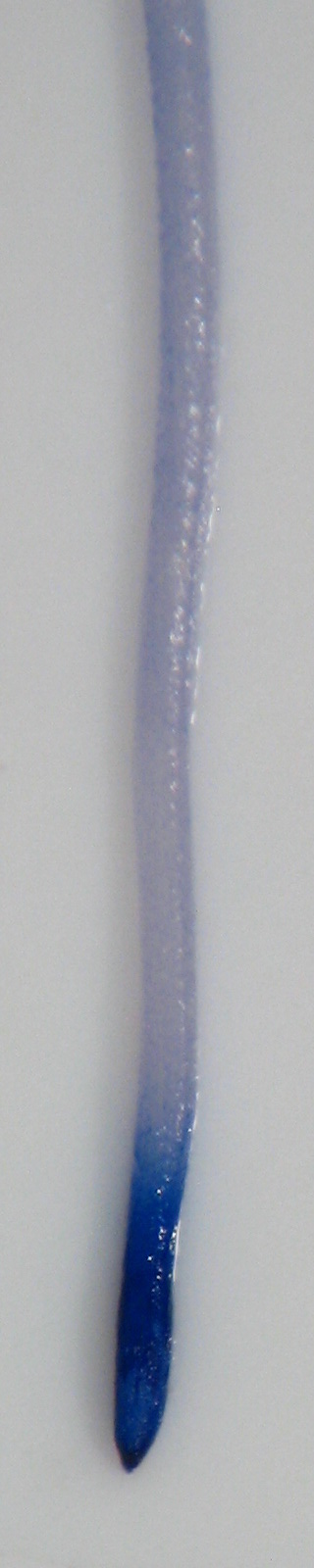

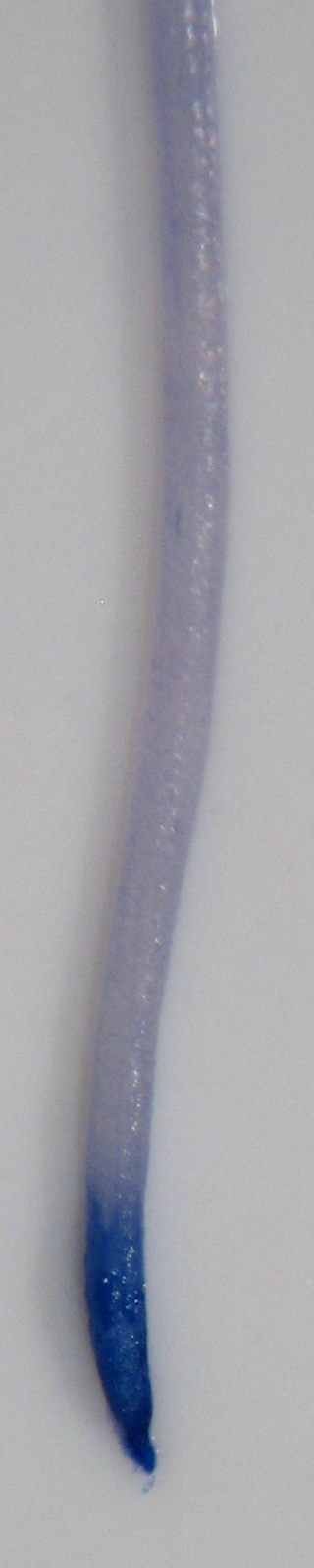

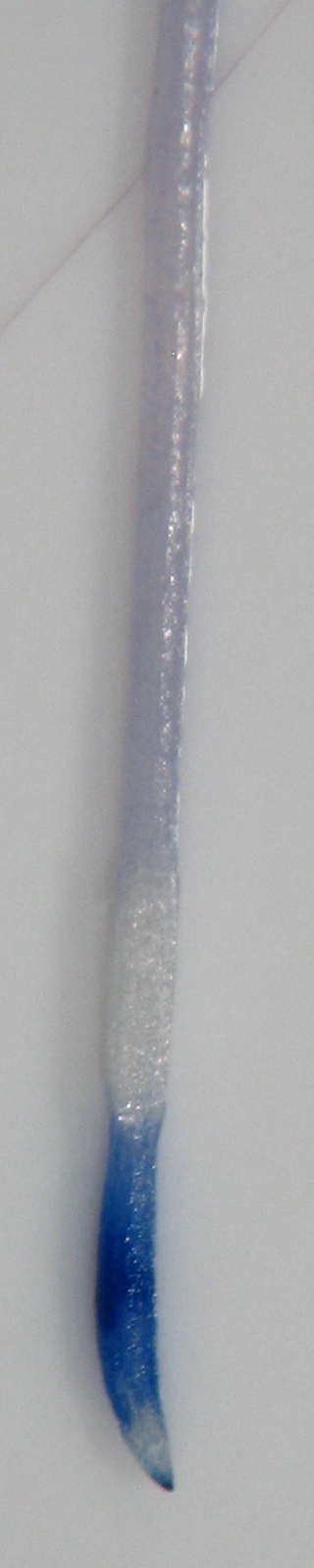

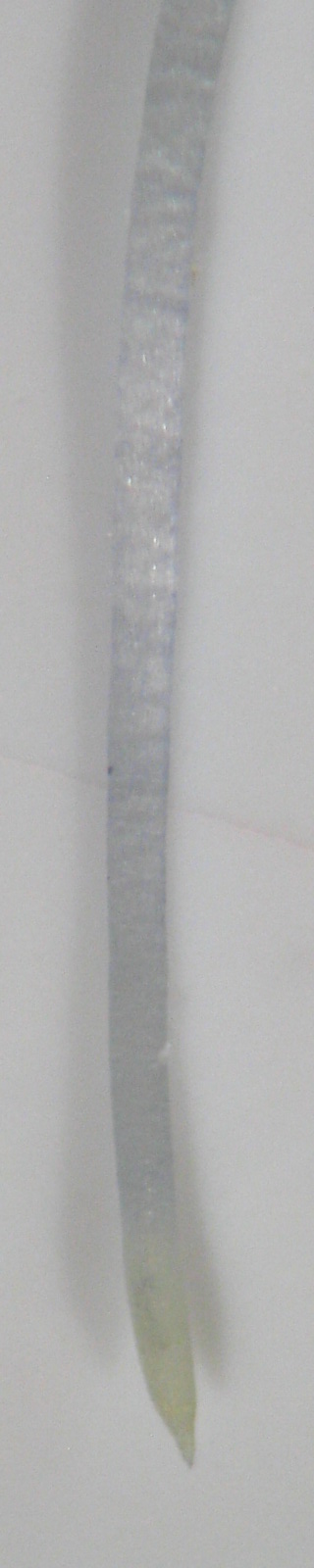

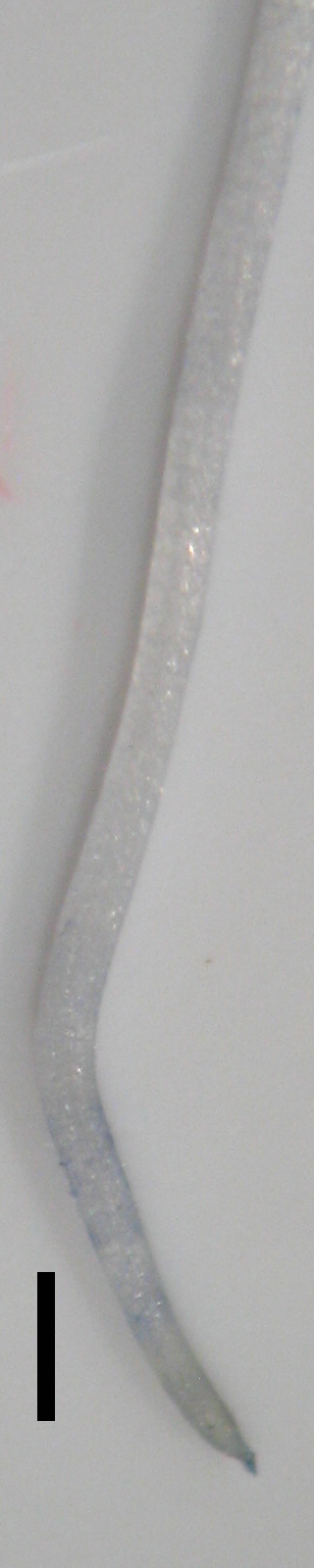

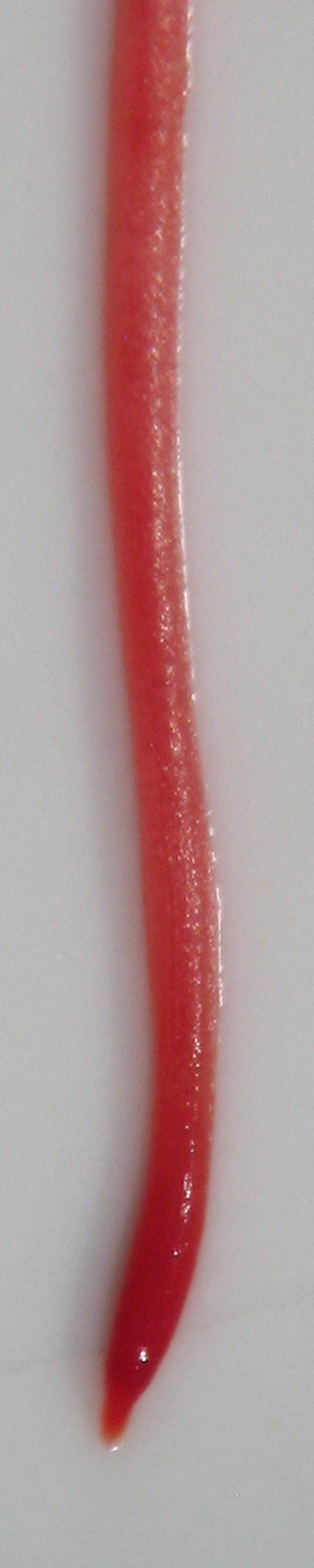

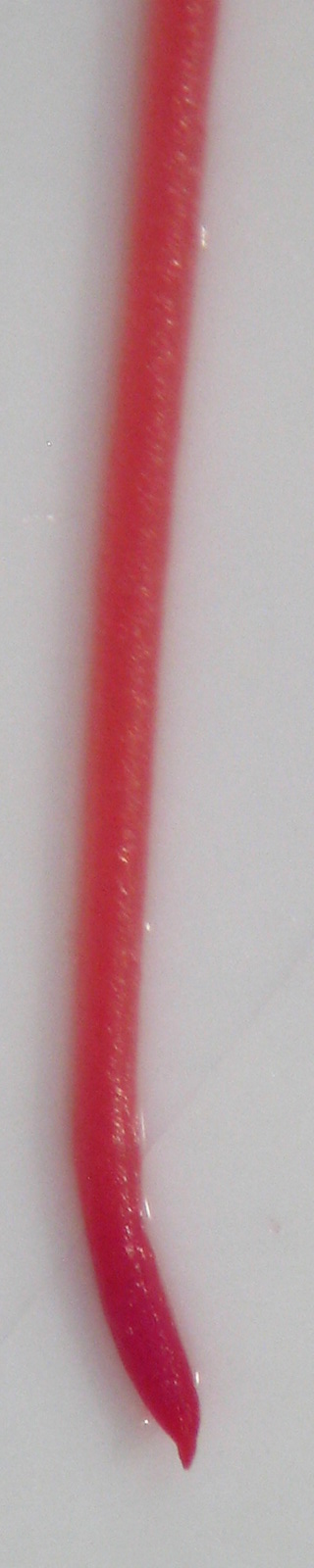

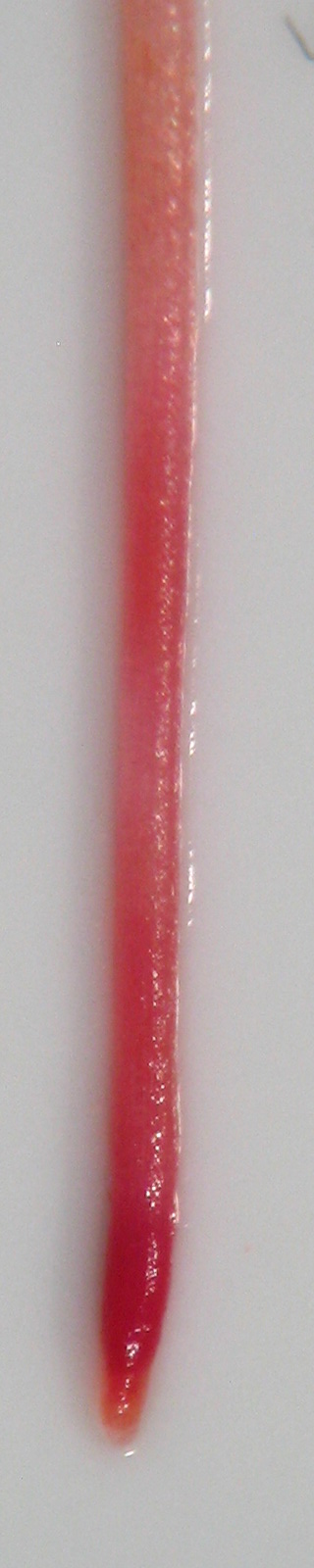

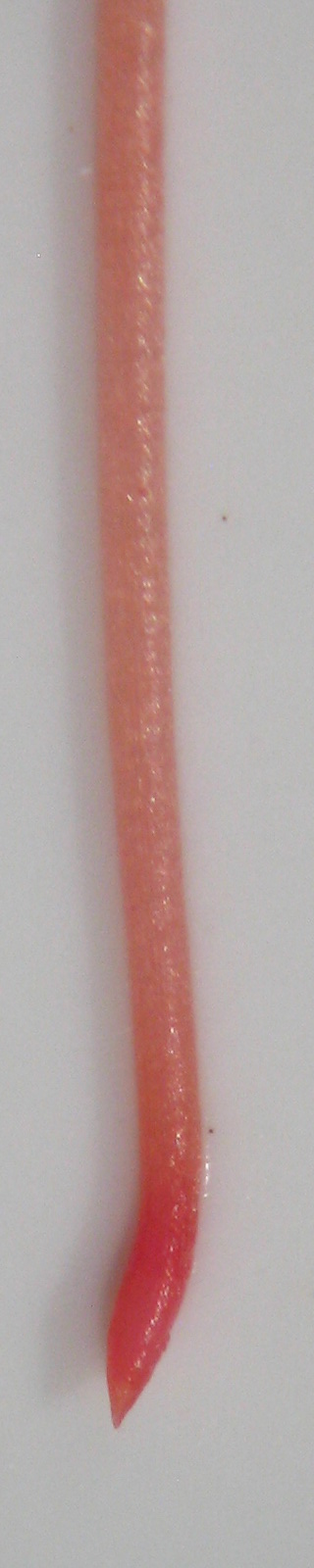

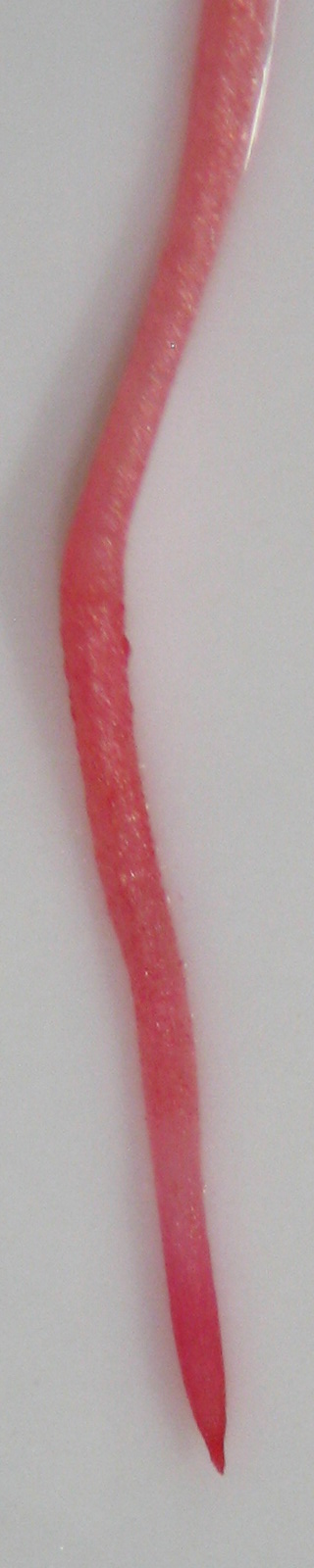

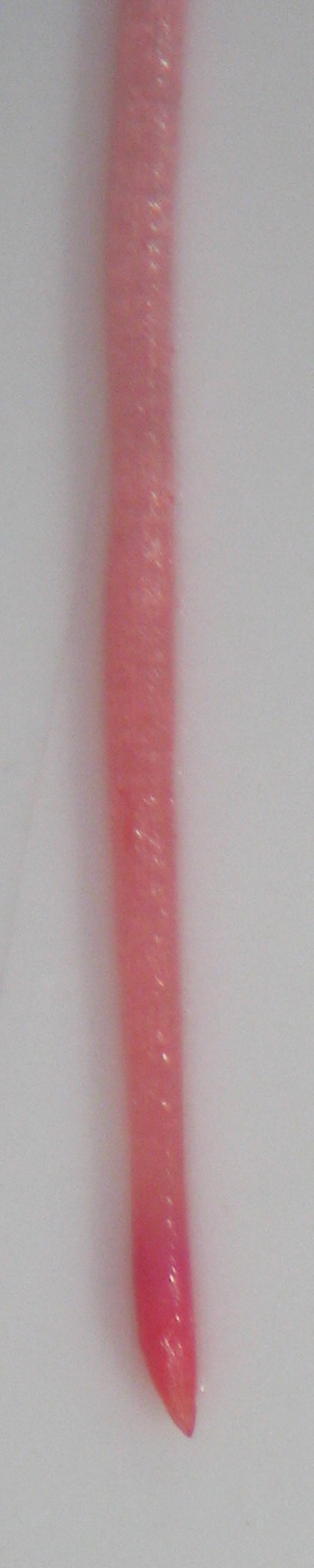

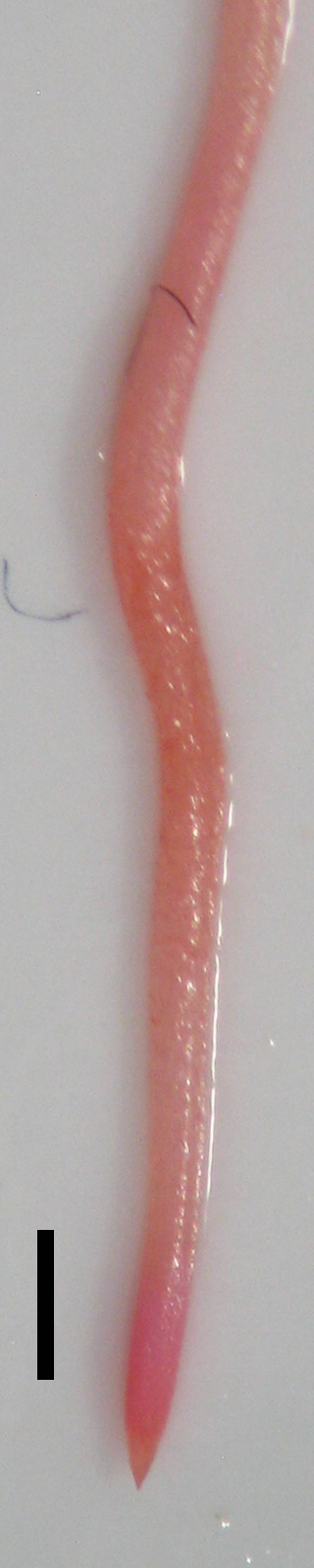


**-Cd**

**-Cd**

**+Cd**

B

**Schiff’s reagent**

A

C

**Evans blue**
