# Supplementary material for: Cadmium-Induced Hydrogen Sulfide Synthesis Is Involved in Cadmium Tolerance in Medicago sativa by Reestablishment of Reduced (Homo)glutathione and Reactive Oxygen Species Homeostases
Source: PLoS One. 2014 Oct 2;9(10):e109669. doi: 10.1371/journal.pone.0109669 (PMC4183592; doi:10.1371/journal.pone.0109669)
Supplement: Figure S2 — H2S or HS−, but not other compounds derived from NaHS contribute to NaHS responses. (DOC) [file pone.0109669.s002.doc]

**Supplementary Figure S2**

**Supplementary Figure S2.** **H_2_S or HS^-^, but not other compounds derived from NaHS contribute to NaHS responses.** Five-day-old seedlings were pretreated with 100 μM NaHS, 100 μM Na_2_S, 100 μM Na_2_SO_4_, 100 μM Na_2_SO_3_, 100 μM NaHSO_4_, 100 μM NaHSO_3_, and 100 μM NaAc for 6 h, and then exposed to 200 μM CdCl_2_ for 72 h (A) or 24h (B). The sample without chemicals was the control (Con). Afterwards, TBARS contents (A) and growth inhibition of 10 seedling roots (B) were determined. Values are means ± SD of three independent experiments with three replicates for each. Bars denoted by the same letter did not differ significantly at *P* < 0.05 according to Duncan’s multiple range test.

B

A
