# Supplementary material for: Cadmium-Induced Hydrogen Sulfide Synthesis Is Involved in Cadmium Tolerance in Medicago sativa by Reestablishment of Reduced (Homo)glutathione and Reactive Oxygen Species Homeostases
Source: PLoS One. 2014 Oct 2;9(10):e109669. doi: 10.1371/journal.pone.0109669 (PMC4183592; doi:10.1371/journal.pone.0109669)
Supplement: Figure S3 — Effects of NaHS, PAG and GSH pretreatments on the fresh weight (A) and TBARS concentrations (B) in alfalfa seedling roots upon Cd stress. (DOC) [file pone.0109669.s003.doc]

**Supplementary Figure S3**

A

B

**Supplementary Figure S3.** **Effects of NaHS, PAG and GSH pretreatments on the fresh weight (A) and TBARS concentrations (B) in alfalfa seedling roots upon Cd stress.** Five-day-old seedlings were pretreated with or without 100 μM NaHS, 2 mM PAG, 1 mM GSH, individual or combination for 6 h, and then exposed to 200 μM CdCl_2_ for 72 h (A) and 24 h (B). Values are means ± SD of three independent experiments with three replicates for each. Bars denoted by the same letter did not differ significantly at *P* < 0.05 according to Duncan’s multiple range test.
