# Supplementary material for: Cadmium-Induced Hydrogen Sulfide Synthesis Is Involved in Cadmium Tolerance in Medicago sativa by Reestablishment of Reduced (Homo)glutathione and Reactive Oxygen Species Homeostases
Source: PLoS One. 2014 Oct 2;9(10):e109669. doi: 10.1371/journal.pone.0109669 (PMC4183592; doi:10.1371/journal.pone.0109669)
Supplement: Figure S4 — Effects of NaHS, PAG and GSH pretreatments on Cd concentrations in alfalfa seedlings upon Cd stress. (DOC) [file pone.0109669.s004.doc]

**Supplementary Figure S4**

**Supplementary Figure S4. Effects of NaHS, PAG and GSH pretreatments on Cd concentrations in alfalfa seedlings upon Cd stress.** Seedlings were pretreated with or without 100 μM NaHS, 2 mM PAG, 1 mM GSH, individual or combination for 6 h, and then exposed to 200 μM CdCl_2_ for 72 h. The sample without chemicals was the control (Con). Values are means ± SD of three independent experiments with three replicates for each. Within each set of experiments, bars denoted by the same letter did not differ significantly at *P* < 0.05 according to Duncan’s multiple range test. ND, none detected.

**Materials and methods**

Determination of Cd content in plant tissues

Cadmium in above-ground parts and root tissues from 240 seedlings were extracted and measured by ICP-OES (Inductively coupled plasma optical emission spectroscopy; Perkin Elmer Optima 2100DV).
