# Supplementary material for: Cadmium-Induced Hydrogen Sulfide Synthesis Is Involved in Cadmium Tolerance in Medicago sativa by Reestablishment of Reduced (Homo)glutathione and Reactive Oxygen Species Homeostases
Source: PLoS One. 2014 Oct 2;9(10):e109669. doi: 10.1371/journal.pone.0109669 (PMC4183592; doi:10.1371/journal.pone.0109669)
Supplement: Table S1 — The sequences of primers for real-time RT-PCR. (DOC) [file pone.0109669.s005.doc]

**Supplementary Table S1.** The sequences of primers for real-time RT-PCR.

| Primer name | Sequences (5’→3’) |
| --- | --- |
| *ECS-F* | CCTTCGGGTTTGAGCAG |
| *ECS-R* | AGCCTAACCTCGGGAAAT |
| *GS-F* | CTGTCAAATGCCCTTCAATA |
| *GS-R* | TGTTTCCTCCTCCTTCTCTC |
| *GR1-F* | TGTGTCATTCGTGGTTGTG |
| *GR1-R* | ACCCGCTATCTTTCCCTC |
| *Cu,Zn-SOD-F* | TAATTGCTGATGCCAACG |
| *Cu,Zn-SOD-R* | ACCACAGGCTAATCTTCCAC |
| *APX1-F* | TCCTCTTATGCTCCGTTTG |
| *APX1-R* | GTTCCACCCAGTAATCCCA |
| *GPX-F* | CGATTTCCTAAGGCTGTTTTG |
| *GPX-R* | AAGTGTGAGAGTTCCGTGTAGTTTG |
| *MSC27-F* | AGAATGGAATGTTGTGGGAGG |
| *MSC27-R* | GTCATCAACACCCTCATCTTCTC |
| *Actin2-F* | AAAAGGATGCCTATGTTGGTG |
| *Actin2-R* | AAGTGGAGCCTCAGTTAGAAGTA |
